# Neutrophil Gelatinase-associated Lipocalin Significantly Correlates with Ischemic Damage in Patients Undergoing Laparoscopic Partial Nephrectomy

**DOI:** 10.4274/balkanmedj.galenos.2018.2018.0852

**Published:** 2019-02-28

**Authors:** Meltem Savran Karadeniz, Isbara Alp Enişte, Hayriye Şentürk Çiftçi, Sebahat Usta, Tzevat Tefik, Öner Şanlı, Kamil Pembeci, Kamil Mehmet Tuğrul

**Affiliations:** 1 Department of Anesthesiology, İstanbul University İstanbul School of Medicine, İstanbul, Turkey; 2 Clinic of Anesthesiology, Grup Florence Nightingale Hospital, İstanbul, Turkey; 3 Department of Medical Biology, İstanbul University İstanbul School of Medicine, İstanbul, Turkey; 4 Department of Urology, İstanbul University İstanbul School of Medicine, İstanbul, Turkey

**Keywords:** Acute kidney injury, laparoscopic partial nephrectomy, neutrophil gelatinase-associated lipocalin, reperfusion damage

## Abstract

**Background:**

Laparoscopic partial nephrectomy, which minimizes renal function loss due to its nephron sparing nature, has become a standard technique among many experienced centers worldwide for surgical treatment of localized kidney tumors. Although partial nephrectomy will remain the gold standard, we need to improve perioperative management and surgical method to prevent postoperative acute kidney injury.

**Aims:**

To demonstrate the frequency of the development of postoperative acute kidney injury following laparoscopic partial nephrectomy in patients with healthy contralateral kidney and determine the early predictive effects of serum neutrophil gelatinase-associated lipocalin on ischemia-reperfusion injury and its association with warm ischemia time.

**Study Design:**

Cross-sectional study.

**Methods:**

Eighty patients were included. We analyzed tumor size, operating time, duration of anesthesia, and warm ischemia time. Serum samples were obtained for measurement of serum creatinine, estimated glomerular filtration rate, and neutrophil gelatinase-associated lipocalin level preoperatively, at the postoperative 2^nd^ hour, and on postoperative days 1 and 2. We used receiver operating characteristic curve for determining the cut-off point of neutrophil gelatinase-associated lipocalin to detect postoperative acute kidney injury. Correlation analysis was performed using Spearman’s test.

**Results:**

Twenty-seven patients developed acute kidney injury on postoperative day 2, and the neutrophil gelatinase-associated lipocalin level increased significantly at the postoperative 2^nd^ hour in the acute kidney injury group (p=0.048). For a cut-off of 129.375 ng/mL neutrophil gelatinase-associated lipocalin, the test showed 70.0% sensitivity and 68.3% specificity for the detection of acute kidney injury at the postoperative 2^nd^ hour. For a cut-off of 184.300 ng/mL neutrophil gelatinase-associated lipocalin, the test exhibited 73.3% sensitivity and 63.3% specificity for the detection of acute kidney injury on postoperative day 1. A significant correlation was found between warm ischemia time and neutrophil gelatinase-associated lipocalin level at the postoperative 2^nd^ hour (r=0.398, p=0.003). The creatinine values were significantly higher and the estimated glomerular filtration rates were significantly lower on postoperative days 1 and 2 in the acute kidney injury group compared with those in the non-acute kidney injury group (p<0.001).

**Conclusion:**

The neutrophil gelatinase-associated lipocalin may be used as an alternative biomarker to serum creatinine in differentiation of ischemic damage in patients undergoing laparoscopic partial nephrectomy.

Laparoscopic partial nephrectomy (LPN), which minimizes renal function loss due to its nephron sparing nature, has become a standard technique among many experienced centers worldwide for surgical treatment of localized kidney tumors (1). Renal artery clamping is used during partial nephrectomy to improve the visibility of the excised surgical field. However, resultant renal ischemia can cause damage to the kidney parenchyma. Several previous reports established that the duration of warm ischemia time (WIT) during cross clamping of renal artery is correlated with the magnitude of renal damage (2,3). Traditionally, WIT should be 20-25 minutes and not exceed 30 minutes (4). Researchers have yet been questioning whether kidney functions are affected in the long-term due to the duration of warm ischemia. Recent studies have demonstrated that maintaining the quality and quantity of renal parenchyma during the surgery is more important in identifying long-term kidney outcomes than the duration of renal ischemia (5,6,7). In addition, the opposite kidney is more sensitive to ischemia in patients with two kidneys relative to patients with solitary kidneys (1). Several diseases, such as diabetes mellitus, hypertension, and coronary artery diseases, may contribute to the immensity of lost renal function after partial nephrectomy (4).

Acute kidney injury (AKI) is the deterioration of kidney functions within hours and is more directly associated with morbidity and mortality than expected in perioperative period. AKI, a significant risk factor for long-term chronic kidney dysfunction, is associated with the development of perioperative increased incidence of sepsis, anemia, coagulopathy, and extended mechanical ventilation and induces the occurrence of injuries in different organs (8,9,10,11). The diagnosis of kidney injury in the early period enables the development of various renoprotective strategies, such as extensive perioperative hemodynamic follow-up and proper fluid regimen regulation. Serum creatinine (sCr) is a commonly used measure of kidney function. However, sCr may be misleading due to differences in age, sex, and body mass index among patients. The sCr levels increase as early as 24-48 hours after the development of kidney injury. In this regard, researchers have used other specific biomarkers for identification of kidney injury to make early diagnosis (12,13,14). Neutrophil gelatinase-associated (NGAL) is a significant biomarker that has diagnostic and prognostic significance in early diagnosis of kidney ischemia–reperfusion injury. We selected NGAL as biomarker in this study because of its high specificity and sensitivity in AKI (15).

In this study, we aimed to demonstrate the frequency of the development of AKI following LPN in patients with healthy contralateral kidney and determine the early predictive effects of serum NGAL levels on ischemia–reperfusion injury and its association with WIT. Our primary hypothesis is that serum NGAL levels would increase in patients with AKI at the postoperative 2^nd^ hour.

## MATERIALS AND METHODS

This study sought the ethics committee approval and obtained written informed consent from the participants. Eighty patients classified with physical status I–III based on the American Society of Anesthesiologists, aged between 40 and 70 years, and scheduled for elective LPN were enrolled in this prospective cross-sectional study between September 2013 and February 2015. Patients were excluded if they had pre-existing renal insufficiency [estimated glomerular filtration rate (eGFR) <60 mL/min/1.73], congestive heart failure, and peripheral vascular disease and if they used nephrotoxic drugs, such as aminoglycosides, amphotericin, and ciclosporin.

### Anesthesia technique

The preoperative preparations and anesthesia techniques were performed similarly among all patients. Routine monitorization was conducted using electrocardiogram, non-invasive blood pressure, and pulse oximetry (SpO_2_). Subsequently, 5 mL/kg/h ringer lactate intravenous infusion was initiated. General anesthesia was induced using 0.03 mg/kg midazolam, 2 mg/kg propofol, 0.6 μg/kg rocuronium, and 0.2 μg/kg remifentanil. Mechanical ventilation was achieved with tidal volume of 8 mL/kg, respiratory rate of 10-12/minute, and positive end-expiratory pressure of 4 cm of H_2_O after the insertion of an endotracheal intubation tube. The patients were positioned at 45°-60° modified flank position with minimal elevation of the kidney bridge. Anesthesia was maintained with 4%-6% desflurane in a mixture of 40% oxygen and 60% air and 0.1 μg/kg/min remifentanil infusion to maintain the intraoperative blood pressure within 20% of the preoperative values. Volume replacement and 5 mg incremental ephedrine were administered to the patients in incidents of hypotension. Bradycardia (heart rate <50 beats/min) was treated using 0.01 mg/kg atropine. At the end of the surgery, the patients were extubated and transported to the postanesthesia care unit.

### Surgery technique

The patients were placed on modified flank position for LPN. About 15 mmHg pneumoperitoneum was created using a Veress needle. Following the insertion of three ports, the bowel was medially mobilized, the ureter was identified, and the renal artery was found. Gerota’s fascia was dissected, and the resection site was scored with monopolar cautery. The tumor was resected on the previously scored margins by using cold scissors following the hilar renal artery clamping. Inner layer renorrhaphy was performed using unidirectional-barbed suture, and the outer layer was closed with sliding Hem-o-lok clip technique. Whenever needed, the defect was covered with oxidized cellulose (Surgicel, Ethicon Inc., Somerville, NJ, USA). The renal artery was unclamped following the completion of renorrhaphy, and the specimen was retrieved with a laparoscopic entrapment bag.

### Clinical outcomes

We analyzed the demographic characteristics of patients, tumor size, operating time, duration of anesthesia, and WIT. Intraoperative hemodynamic data (heart rate and mean arterial pressure) were recorded. The levels of sCr, eGFR, and NGAL were evaluated before the surgery (preoperative), 2 hours after completion of the surgery (postoperative 2^nd^ hour), and on postoperative days 1 and 2. eGFRs were calculated using the “chronic kidney disease epidemiology” formula (16). Postoperative AKI was defined as an increase in the sCr level within the postoperative 48 hours by more than or equal to 50% from the baseline (17).

### Collection of serum samples

Blood samples were obtained for measurement of sCr, eGFR, and NGAL levels. The samples were collected preoperatively, at the postoperative 2^nd^ hour, and on postoperative days 1 and 2. Peripheral venous blood samples were collected in vacutainers and allowed to clot for 30 minutes at room temperature prior to centrifugation at 1.600×g for 10 minutes at room temperature. The samples were stored at -80 °C until assay.

### Determination of serum NGAL levels

NGAL concentration was determined using an enzyme-linked immunosorbent assay kit (Biovendor–Laboratorni Medicina, Brno, Czech Republic). NGAL detection kits were labeled for research use and incorporated in assays based on sandwich enzyme-linked immunosorbent assay technology. Absorbance was recorded with a microtiter plate reader at 450 nm. Each sample was tested in duplicate, and the coefficient of intraassay variation among the duplicates was <10%. NGAL concentration was calculated from the standard curves by linear regression analysis.

### Statistical analysis

Statistical analysis was performed using the Statistical Package for the Social Sciences version 21.0. The creatinine level was 1.01±0.4 mg/dL on postoperative day 2 in patients with AKI in our pilot study. A minimum of 70 patients would be required to obtain a 0.28 difference with a standard deviation of 0.4, with α and β errors of 0.05 and 0.2, respectively. Therefore, 80 patients were selected in case of drop outs. eGFR was calculated by “chronic kidney disease epidemiology” formula. Kolmogorov–Smirnov test was performed to assess deviation from normal distribution. Quantitative variables were summarized as mean and standard deviation or as median. The different patient groups (AKI group vs non-AKI group) were compared using Student’s t-test and Mann-Whitney U test for quantitative variables and X^2^ test for categorical data. Differences among all parameters at different time points were assessed by repeated measures ANOVA for quantitative variables. Correlation analysis was performed using Spearman’s test. Factors potentially influencing variations in NGAL level were evaluated in univariate analysis. The threshold value of NGAL level in accordance with AKI classification was investigated using receiver operating characteristic. Sensitivity, specificity, positive predictive level, negative predictive level, and accuracy rate were evaluated in accordance with the specified threshold levels. The significance value was regarded as p<0.05.

## RESULTS

**Figure 1:**
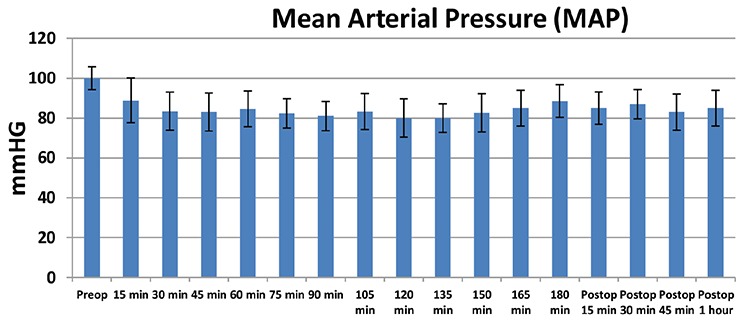
Mean arterial pressure (mmHg) follow-up throughout the perioperative period. Data are presented as mean ± standard deviation.

**Figure 2:**
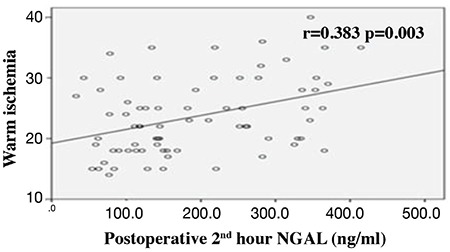
Correlation of warm ischemia time and NGAL levels at postoperative 2^nd^ hour. NGAL: neutrophil gelatinase-associated lipocalin

**Figure 3:**
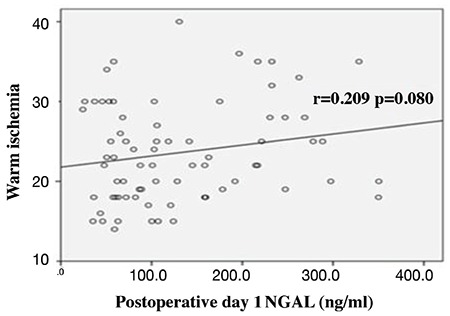
Correlation of warm ischemia time and NGAL levels at postoperative day 1. NGAL: neutrophil gelatinase-associated lipocalin

Eighty patients who underwent LPN were included in the study. Five patients were excluded because their serum samples were not stored under the optimal conditions. The demographic and clinical characteristics of the 75 patients are shown in Table 1. The patients were divided into AKI and non-AKI groups. We did not observe any significant differences in demographic data between the two groups (p>0.05). Baseline (preoperative), sCr, eGFR, and NGAL levels were not significantly different between AKI and non-AKI groups (p>0.05) (Table 1). No significant difference was also observed in mean arterial pressure values during the intraoperative and postoperative early period (p>0.05) (Figure 1). The sCr values were significantly higher and the eGFR values were significantly lower on postoperative days 1 and 2 compared with the preoperative values (p<0.001) (Table 2). The NGAL levels were significantly higher at the postoperative 2^nd^ hour and on postoperative day 1 compared with the preoperative values (p<0.001) (Table 2). We analyzed the correlation among the variables measured. A significant correlation was found between WIT and NGAL levels at the postoperative 2^nd^ hour (r=0.398, p=0.003) (Figure 2). However, this significance disappeared on postoperative day 1 (r=0.209, p=0.081) (Figure 3). Twenty-seven patients developed AKI on postoperative day 2. The NGAL levels increased significantly at the postoperative 2^nd^ hour in the AKI group compared with those in the non-AKI group (p=0.048). However, on postoperative day 1, no significant difference was found between AKI and non-AKI groups (p>0.05) (Table 3). The sCr values were significantly higher and the eGFR values were significantly lower on postoperative days 1 and 2 in the AKI group compared with those in the non-AKI group (p<0.001) (Table 3). Twenty-four patients had NGAL levels above the calculated cut-off value despite having normal sCr levels on postoperative day 1.

**Figure 4:**
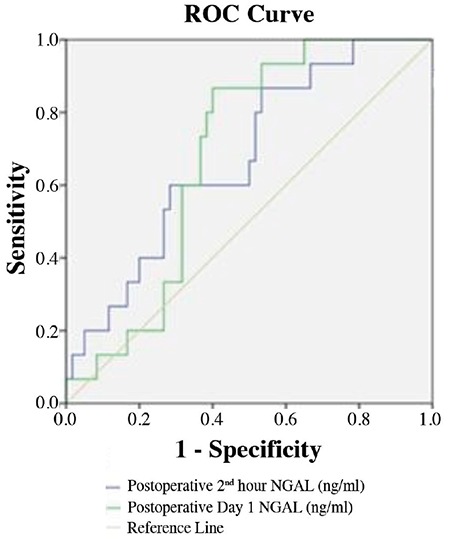
ROC curve of NGAL. Sensitivity, specificity, positive and negative predictivity of NGAL levels at postoperative 2^nd^ hour and on postoperative day 1 in patients who developed AKI. NGAL: neutrophil gelatinase-associated lipocalin; ROC: receiver-operating characteristic; AKI: acute kidney injury

We determined the sensitivities, specificities, and positive and negative predictive values for NGAL at different cut-off levels for the postoperative 2^nd^ hour and on postoperative day 1 (Tables 4, 5). Figure 4 shows the receiver operating characteristic curve for the postoperative NGAL levels. The area under the curve could be explained as follows.

The area under the curve was 0.627 [confidence interval (CI): 0.494-0.759, p=0.042]; at a cut-off value of 129.375 ng/mL NGAL at the postoperative 2^nd^ hour, the sensitivity and specificity were 70.0% and 68.3%, respectively. The receiver operating characteristic curve analysis confirmed the positive value of NGAL for predicting AKI (46.5%) at the postoperative 2^nd^ hour (Table 4).

The area under the curve was 0.623 (CI: 0.489-0.758, p=0.048); and at a cut-off value of 184.300 ng/mL NGAL on day 1, the sensitivity and specificity were 73.3% and 63.3%, respectively. The receiver operating characteristic curve analysis confirmed the positive value of NGAL for prediction of AKI (53.3%) on postoperative day 1 (Table 5).

## DISCUSSION

In this prospective clinical study, we observed that the serum NGAL levels significantly increased at the postoperative 2^nd^ hour in patients with AKI compared with those in patients without AKI. A positive correlation was detected between WIT and serum NGAL levels at the postoperative 2^nd^ hour. In addition, the sCr and NGAL levels were significantly higher in the postoperative period than in the preoperative period. We suggested that the rate of postoperative development of AKI might be higher than the estimated rate in such cases and that NGAL could a significant biomarker in early diagnosis of kidney damage.

In partial nephrectomy, the primary goal is to control cancer while preserving the maximal renal function and obtaining minimal morbidity in the perioperative period. We found a decrease in kidney functions by about 20% in solitary operated kidney and by 10% in global function in partial nephrectomies. Progressive chronic kidney failure may also develop in acute period in addition to severe kidney damage that required dialysis in 1% of patients (18).

The development of severe AKI is associated with conditions, such as the need for intensive care and dialysis, which increases hospital stay (10). The most significant factor in preservation of kidney function in minimal invasive nephrectomies is the preservation of the nephron mass. Tumor size, loss of normal parenchyma during tumor resection, deterioration of blood flow, and blood saturation cause renal function loss in the postoperative period (19,20). Mir et al. (21) reported a significant decrease in the eGFR value after the resection of huge tumors. Another study on patients with solitary kidney reported that a 15% loss of parenchyma caused deterioration of renal function by 19.7% postoperatively (22). Whether WIT promotes kidney function deterioration remains controversial. In their first study, Thompson et al. (23,24) reported that increased ischemia time caused a decrease in kidney functions. However, in the proceeding follow-up of the same patient group, they reported that WIT had no predictive value in the diagnosis when predicting for long-term renal dysfunction. After the correlative analyses of biomarkers and histologic evaluations, different authors reported that minimal structural and functional changes might develop during the WIT of 15-60 minutes, which would not result in irreversible changes in kidneys (11). In our study, the mean tumor size was 3.6 cm, and the mean WIT was 24 minutes. This period is within the reasonable limits as mentioned in the above studies. In addition, the NGAL increase at the postoperative 2^nd^ hour was positively correlated with WIT.

Hemodynamic changes, particularly mean arterial pressure, in the intraoperative period have a significant role in kidney perfusion. Even the short-period hypotension attacks in this period may cause ischemia in the kidney. Walsh et al. (25) reported the correlation of mean arterial pressure (mean arterial pressure <55 mmHg) with the development of AKI in a wide patient population of non-cardiac surgery group. Mean arterial pressure <60 mmHg for over 20 minutes or mean arterial pressure <55 mmHg for over 10 minutes was associated with high AKI risk (26). No hypotension attack unresponsive to fluid and ephedrine treatment was observed in the intraoperative period in our study. We detected no differences in the periodic measurements during the surgery. Hemodynamic stability is as important as the other factors in influencing ischemia risk after partial nephrectomy.

Energy stocks are wasted due to decrease in enabling oxygen to the tissues after ischemia because of various causes in the kidney; this phenomenon results in cell necrosis and accumulation of toxic metabolites. The products developed after the reperfusion of ischemic tissue activate the mast cells and neutrophils; the local cell damage causes systemic inflammatory reaction and secretion of cytokine and inflammatory mediators that cause multi organ failure (27). Therefore, early diagnosis of AKI, proper monitorization, and initiation of protective treatments are significant measures for prevention of permanent kidney damage. sCr and accompanying eGFR changes in diagnosis of AKI may frequently be missed out particularly when the opposite kidney is healthy (1) and thus could not predict the initial time of damage neither the degree of severity. sCr starts to increase when the kidney function decreases by 50% and varies in accordance with individual factors, such as muscle mass, sex, exercise, and diet. Thus, researchers have explored specific and sensitive new biomarkers to diagnose ischemia in recent years.

NGAL, a member of the lipocalin family, is secreted from kidney proximal tubuli after ischemic damage. Ischemia causes an increase in the urine and serum levels of NGAL. However, NGAL was not routinely used in the clinics but has been employed in cardiovascular surgery, intensive care, and transplant surgery; NGAL increased within 2 hours immediately after the development of damage (28,29). Woodson et al. (12) created warm ischemia for 15, 30, and 60 minutes in rats with a single kidney and demonstrated that the urine NGAL level increased to the maximum level within 30 minutes of renal artery clamping. In the present study, we used serum NGAL value. As indicated by Sprenkle, monitoring the NGAL increase in serum may be less misleading because the increase in the production in ischemic kidney in partial nephrectomies of healthy patients with two functional kidneys might be diluted if the opposite kidney produces a high volume of urine (13).

To date, few clinical studies have demonstrated the importance of NGAL in diagnosis of possible AKI in partial nephrectomies. However, Abbasi et al. (30) reported an increase in the urine NGAL levels at the 1^st^ hour following the cold ischemia in nephron sparing open partial nephrectomies of a small number of patients. Sprenkle found no significant increase in the NGAL levels in the urine after open partial nephrectomy in individuals with healthy kidney functions and did not accept NGAL as a diagnostic factor. The detection of less damage in their study might be due to several factors, such as performing cold ischemia, which has a better kidney protection effect, in most patients during the renal artery clamping, and infusion of intraoperative mannitol (13).

We conducted tumor resection using laparoscopic method and under the warm ischemia in all patients. Pneumoperitoneum during laparoscopy increases the intraabdominal pressure, which might contribute to the deterioration of kidney perfusion. Similar to our study, the results of the study of Koo et al. (31) showed increased NGAL levels in the urine at the early postoperative period after LPN in patients with healthy contralateral kidney. However, they reported that the increased urine NGAL had no predictive value in the determination of the severity of AKI and in the diagnosis of long-term renal dysfunction.

In this study, the diagnostic value of serum NGAL level was measured using the receiver operating characteristic curve. The receiver operating characteristic curve analysis showed that serum NGAL level has a valuable diagnostic performance. As evidenced by the area under the curve of 0.67, the biomarker NGAL has a good ability to predict AKI in the first 2 hours after partial nephrectomy.

In our study, the serum NGAL level was significantly higher at the 2^nd^ hour compared with the preoperative values. The postoperative sCr level was also significantly higher compared with the preoperative values. In addition, AKI developed in 36% of the patients on day 2, and their NGAL levels in the early period were significantly higher. Due to the lack of information about long-term patient follow-up, the rate of permanent renal damage is difficult to predict. This aspect can be regarded as a limitation of our study.

In conclusion, this study showed that increased serum NGAL levels at the 2^nd^ hour post LPN are predictive of increased risk of AKI. Early diagnosis of renal injury may prevent the side effects of this undesirable condition. Despite that it is more expensive than sCr, NGAL is an alternative biomarker of AKI in the early postoperative period especially in patients with long WIT.

## Figures and Tables

**Table 1:**
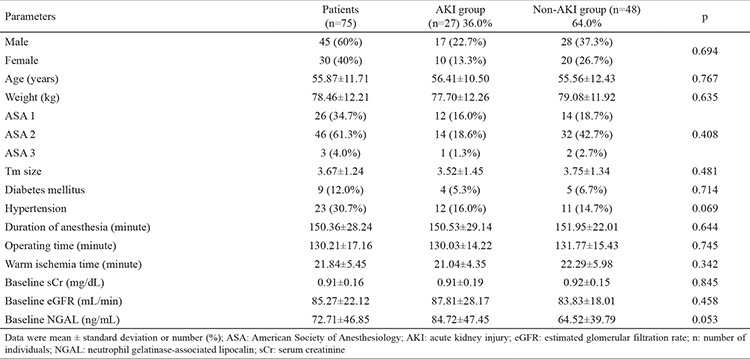
Demographic characteristics of patients

**Table 2:**
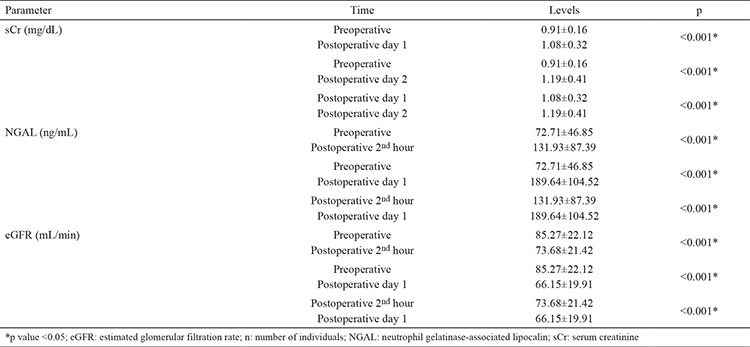
Levels of sCr, NGAL, and eGFR presented as mean ± standard deviation

**Table 3:**
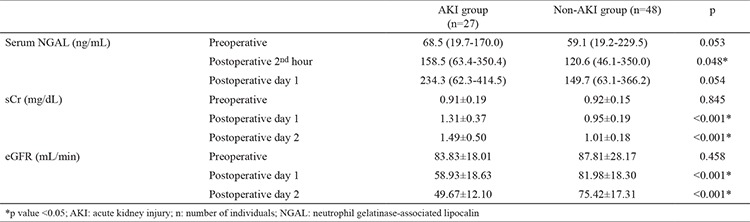
NGAL presented as median (minimum-maximum) and sCr and eGFR presented as mean ± standard deviation

**Table 4:**
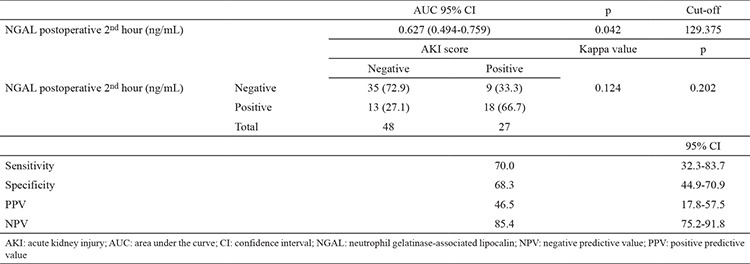
Sensitivity, specificity, and positive and negative predictive values of NGAL levels at the postoperative 2^nd^ hour in patients who developed AKI

**Table 5:**
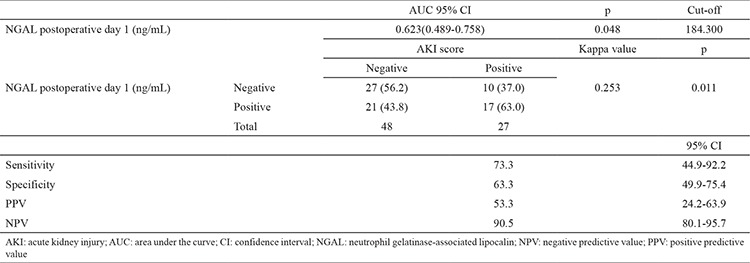
Sensitivity, specificity, and positive and negative predictive values of NGAL levels on postoperative day 1 in patients who developed AKI
